# Mitochondrial Respiration Is Decreased in Rat Kidney Following Fetal Exposure to a MaternalLow-ProteinDiet

**DOI:** 10.1155/2012/989037

**Published:** 2012-03-27

**Authors:** Sarah Engeham, Kennedy Mdaki, Kirsty Jewell, Ruth Austin, Alexander N. Lehner, Simon C. Langley-Evans

**Affiliations:** ^1^Mitochondrial Research Group, Institute for Ageing and Health, Newcastle University, Campus for Ageing and Vitality, Newcastle upon Tyne NE4 5PL, UK; ^2^School of Health, The University of Northampton, Park Campus, Boughton Green Road, Northampton NN2 7AL, UK; ^3^School of Biosciences, University of Nottingham, Sutton Bonington, Loughborough LE12 5RD, UK

## Abstract

Maternal protein restriction in rat pregnancy is associated with impaired renal development and age-related loss of renal function in the resulting offspring. Pregnant rats were fed either control or low-protein (LP) diets, and kidneys from their male offspring were collected at 4, 13, or 16 weeks of age. Mitochondrial state 3 and state 4 respiratory rates were decreased by a third in the LP exposed adults. The reduction in mitochondrial function was not explained by complex IV deficiency or altered expression of the complex I subunits that are typically associated with mitochondrial dysfunction. Similarly, there was no evidence that LP-exposure resulted in greater oxidative damage to the kidney, differential expression of ATP synthetase **β**-subunit, and ATP-ADP translocase 1. mRNA expression of uncoupling protein 2 was increased in adult rats exposed to LP *in utero*, but there was no evidence of differential expression at the protein level. Exposure to maternal undernutrition is associated with a decrease in mitochondrial respiration in kidneys of adult rats. In the absence of gross disturbances in respiratory chain protein expression, programming of coupling efficiency may explain the long-term impact of the maternal diet.

## 1. Introduction

Retrospective associations between low weight or thinness at birth and risk of cardiovascular disease and type 2 diabetes gave rise to the hypothesis that maternal nutritional status may be one of a number of factors that programme long-term risk of disease [1–4].This hypothesis has received strong support from studies of small and large animal species, which overwhelmingly indicate that exposure to undernutrition in pregnancy, whether specific to macronutrients [5] or micronutrients (e.g., iron) [6], or in the form of lower overall food intake [7], programmes risk of adult hypertension, glucose intolerance, insulin resistance, and dyslipidaemia [5–9].Maternal protein restriction during rat pregnancy has been widely reported to impact upon blood pressure in the exposed offspring and the hypertension observed in such animals has partly been linked to programming of renal development [10].

Maternal protein restriction brings about early-life programming through remodelling of specific tissues [11]. In the kidney, the number of nephrons is reduced through exposure to maternal undernutrition, and with ageing, low-protein-exposed animals develop glomerular injury and progressive loss of renal function [10, 12, 13]. There is evidence to suggest that this is, at least in part, mediated by decreased activity of antioxidant enzymes and an associated increase in oxidative injury [13, 14]. The basis for these effects of maternal diet is unclear and whilst programming is widely believed to be driven by epigenetic mechanisms [15], there is no specific evidence that there is differential regulation of genes which determine antioxidant-reactive oxygen species balance. One hypothesis is that the programming is achieved through altered regulation of coupling efficiency.

Mitochondrial respiration is the major source of reactive oxygen species in mammalian tissues, with superoxide radicals emanating from the respiratory chain at several points, most notably the transfer of electrons by complexes I and III. Mitochondrial dysfunction has been linked to many of the diseases also associated with early-life programming, including atherosclerosis, hypertension, renal dysfunction, and diabetes [16]. There are some reports that mitochondrial dysfunction may play a role in the declining physiological function in animals exposed to less than optimal maternal nutrition during fetal life. Most studies have considered the impact of maternal overnutrition. Offspring of mice rendered obese prior to pregnancy exhibit functional deficits in mitochondrial function at the early embryonic stage and postnatal [17]. Such changes are associated with an increase in mitochondrial DNA copy number and reactive oxygen species generation. In rats exposed to a maternal high-fat diet, kidney mitochondrial DNA was decreased, and expression of the mitochondrial genome in aorta was downregulated [18]. Mortensen and colleagues reported that mice exposed to maternal protein restriction showed changes in expression of mitochondrial genes, with differential responses to diet in liver and skeletal muscle [19]. However, no functional phenotype was described in this study. Together such findings suggest that mitochondria may be targets for programming in response to a number of insults, but to date no study has integrated a study of programmed changes in gene or protein expression with measures of mitochondrial function in the kidney. The aim of this study was therefore to explore the hypothesis that the renal dysfunction that is associated with maternal protein restriction is a consequence of programming of mitochondrial function via alterations in the expression of mitochondrial genes and proteins and to the regulation of coupling efficiency.

## 2. Materials and Methods

### 2.1. Animals

Two separate animal experiments were carried out under license from the UK Home Office, in accordance with the Animals (Scientific Procedures) Act 1986. In both trials, virgin female Wistar rats (Harlan, UK) were mated at between 180 and 225 g weight, with stud males. Upon confirmation of pregnancy by the presence of a semen plug, the female rats were randomly allocated to be fed one of two isocaloric synthetic diets (18% casein, CON; 9% casein, LP), as reported previously [20, 21]. In the first experiment 10 pregnant rats were fed the semi-synthetic diets (5 CON; 5 LP) until they delivered pups at 22d gestation. Upon delivery of pups, the mothers were transferred to a standard laboratory chow diet (B&K Universal Ltd, Hull, UK, rat and mouse diet) and the litters were culled to a maximum of 8 pups, (4 males and 4 females). This minimized variation in nutrition during the suckling period. The offspring from the two groups therefore differed only in terms of their prenatal dietary experience. At 16 weeks after birth, 2 male animals from each litter were culled using carbon dioxide asphyxia and cervical dislocation. For one animal in each litter, the right kidney was collected, snap-frozen in liquid nitrogen, and stored at −80°C for later analysis of gene expression. Both kidneys were collected from a second male from each litter, and these were used for fresh preparation of mitochondria and analysis of respiratory chain function. In the second experiment, 12 pregnant rats were fed the semisynthetic diets (6 CON; 6 LP) until they delivered pups at 22d gestation. The protocol for maternal feeding and for weaning the offspring was as described above. At 4 weeks after birth, the pups were weaned onto chow diet and half of the animals were then culled using carbon dioxide asphyxia and cervical dislocation. The right kidney was collected, snap-frozen in liquid nitrogen, and stored at −80°C. The remaining animals were maintained on standard laboratory chow and culled at 13 weeks of age. Kidneys were collected, snap-frozen in liquid nitrogen, and stored at −80°C.

Kidney samples from male offspring used for histochemistry, immunohistochemistry, and analysis of mitochondrial DNA copy number (experiment two) were resnap frozen in isopentane and stored in a −80°C freezer. Serial sections were obtained using a cryostat at −20°C to a thickness of 15 *μ*m and then stored at −80°C after air drying. All sections were air-dried for 90 minutes at room temperature prior to use.

### 2.2. Histochemistry

#### 2.2.1. Complex IV Histochemistry

Mitochondrial respiratory chain activity was determined using sequential complex IV(COX)/complex II (succinate dehydrogenase [SDH]) histochemistry. Complex IV activity was detected using COX medium. Any cells with complex IV activity stained brown. To assess whether there was evidence of complex IV deficiency, sections were washed twice in phosphate-buffered saline and the SDH medium, an indicator of complex II activity, was applied, blue staining any cells that failed to stain brown (indicating complex IV activity). Sections were dehydrated in 70%, 95%, and 100% ethanol and mounted in DPX resin. Images were captured using a Zeiss microscope and analysed using Adobe Photoshop CS4. Areas of mitochondria were highlighted, and intensity within each area was calculated using the measurement tool.

### 2.3. Immunohistochemistry

Immunohistochemistry was used to determine variation in expression of a variety of proteins including some oxidative phosphorylation (OXPHOS) proteins and some markers for oxidative stress. Complex I-19 (CI-19; antibody diluted 1 in 300), Complex I-20 (CI-20; 1 in 50), and complex IV subunit I (1 in 200) are subunits of the OXPHOS complexes that are commonly affected in mitochondrial dysfunction. They are encoded by the mitochondrial genome. Complex II-70 (CII-70; 1 in 300) is rarely affected in mitochondrial dysfunction. It is encoded by the nuclear genome. Phospho-histone H_2_AX (H2AX; 1 in 200) and 8-Hydroxy-2′-deoxyguanosine (8-OHdG; 1 in 200) are markers of oxidative damage to DNA, and superoxide dismutase 2 (SOD2; 1 in 1000) is an antioxidant produced in the mitochondria in response to reactive oxygen species.

All sections for immunohistochemistry were fixed in 4% paraformaldehyde solution for 10 minutes, followed by alcohol permeabilisation. Nonspecific binding was blocked using an avidin biotin blocking kit (Sigma, UK), followed by 1% normal goat serum (Sigma). The sections were incubated with primary antibodies for 90 minutes at room temperature. The secondary antibodies (either antimouse rat adsorbed or antirabbit biotinylated) were incubated for 30 minutes, followed by ABC (Sigma) for a further 30 minutes and DAB (Sigma) for approximately 6 minutes. Sections were then dehydrated and mounted, as for COX/SDH histochemistry. Images were captured using a Zeiss microscope and analysed using Adobe Photoshop CS4.

### 2.4. Mitochondrial DNA Copy Number

For laser microdissection, cryostat sections (15 *μ*m thick) were mounted onto membrane slides (Leica), and SDH histochemistry was performed. To determine mtDNA copy number, tissue (approximately 1000 *μ*m²) was dissected from proximal convoluted tubules using a Leica laser micro-dissection microscope (Leica LMD). DNA extraction was carried out using a lysis buffer containing tris-HCl, tween 20, and proteinase K.

For estimation of mtDNA copy number, the control region (D-loop) of rat mtDNA was amplified using the primers and probe as follows: forward primer (5′-GGT TCT TAC TTC AGG GCC ATC A-3′), reverse primer (5′-GAT TAG ACC CGTTAC CAT CGA GAT-3′), and probe (6FAM-TTG GTT CAT CGT CCA TAC GTT CCC CTT A-TAMRA). The PCR program consisted of a 2 min incubation at 50°C, 10 min at 95°C, and 40 cycles of amplification; 15 sec denaturation at 95°C; 1 min at 60°C for hybridization of probes, primers and DNA synthesis. Mitochondrial DNA copy number was calculated per *μ*m² from a standard curve.

### 2.5. Rat Kidney Mitochondrial Function

Whole kidneys from experiment two were chopped and minced, then homogenised in STE (250 mM sucrose, 5 mM Tris/HCl at pH 7.4, and 2 mM EGTA) medium using a Teflon dounce homogeniser. Mitochondria were prepared by differential centrifugation at 4°C using STE, adapting a method described by Rolfe et al. [22]. The protein concentrations of the mitochondria preparations were determined by the Biuret method with bovine serum albumin (BSA) as a standard [23].

#### 2.5.1. Mitochondrial Respiration

The assay medium consisted of 120 mM KCl, 5 mM KH_2_PO_4_, 3 mM HEPES, 1 mM EGTA, and 0.3% (w/v) BSA at pH 7.2. Respiration was measured inside a sealed Clark oxygen electrode (Rank Brothers Ltd, UK) at a temperature of 37°C. The medium was air-saturated and assumed to contain 406 nmol of oxygen/mL [24]. Electrodes were monitored for linearity from 100% to 0% air saturation for each mitochondrial preparation. Mitochondria were resuspended to 0.35 mg of protein/mL in assay medium containing rotenone (5 *μ*M) and succinate (4 mM). Excess ADP (1 mM) was added to establish state 3 respiration [25]. Maximum state 3 respirations were established by adding carbonyl cyanide p-trifluoromethoxyphenyl-hydrazone, up to 5 *μ*M.

#### 2.5.2. Measurement of Proton Conductance

Triphenylmethylphosphonium (TPMP), the potential sensitive probe was used to measure membrane potential by detecting the external TPMP in the medium. This was done in the presence of rotenone (5 *μ*M), nigericin (80 ng/mL to clamp pH gradient to 0), and oligomycin (at 1 *μ*g/mL to inhibit ATP synthesis). The electrode was calibrated with sequential 1 *μ*M additions up to 5 *μ*M TPMP. Succinate (4 mM) was used to start respiration. Membrane potential and oxygen consumption were progressively inhibited through successive steady states using up to 4 mM Malonate [22]. This was continuously monitored and data displayed on the computerised system that used Chart 5 software (AD instruments, UK). At the end of each experimental run, 0.5 *μ*M FCCP was added to dissipate the membrane potential. This released all internal TPMP into the medium allowing correction for any small electrode drift. TPMP binding correction was assumed to be 0.4 *μ*l/mg protein for kidney [26]. The respiration at each steady state was plotted against the corresponding membrane potential.

### 2.6. Quantitative Real-Time PCR

Quantitative real-time PCR was used to determine mRNA expression, using a Roche LightCycler [27]. Total RNA was extracted from the kidneys (experiment 1) using a TRIzol extraction (Invitrogen, UK). The RNA was DNase-treated (Promega, UK) to remove any genomic DNA and then extracted using a phenol-chloroform extraction followed by ethanol precipitation. Copy DNA (from 0.5 *μ*g RNA) was reversely transcribed using Maloney murine leukemia virus RT (Promega). Template-specific primers were designed for UCP2: (forward primer: GACCTCATCAAAGATACTCCTGAAb and reverse primer: CAATGACGGTGGTGCAGAAG); ATP Synthetase *β*-subunit (forward primer: CCACCAAGAAGGGCTCGAT and reverse primer: GGCAGGGTCAGTTCAGGTCAT); ATP-ADP translocase 1 (forward primer: CCCGATCGAGAGGGTCAAA and reverse primer: TGTACTGTTTCTCTGCACTGATCTGT); the housekeeping gene *β*-actin using Primer Express Version 1.5 (Applied Biosystems, USA). A standard curve was produced using a pool of the copy DNA, and all samples were normalised to *β*-actin expression. *β* -actin expression was not influenced by maternal diet. 

### 2.7. Western Blotting

Expression of UCP2 (primary antibody; Calbiochem, cat. number 662047) and ATP synthetase *β*-subunit (primary antibody; Sigma cat. number A9728) protein was determined in mitochondrial preparations by Western blotting using previously described methods [28]. Expression was normalised to *β*-actin.

### 2.8. Statistics

Student's *t*-test was used to determine statistical significance between maternal dietary groups. All data are shown as mean ± SEM. *P* < 0.05 was accepted as statistically significant.

## 3. Results and Discussion

**Figure 1:**
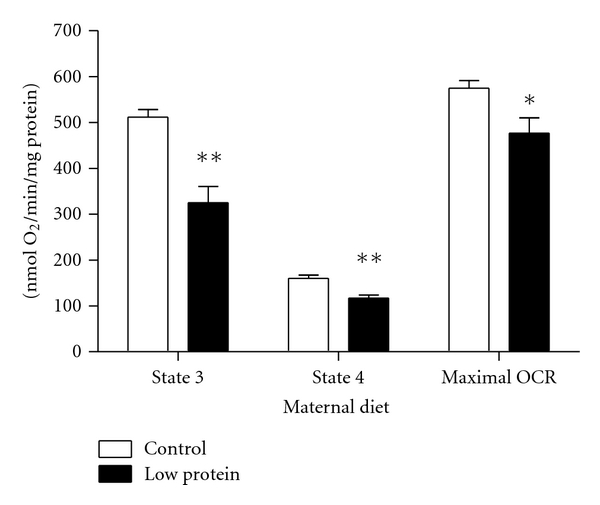
Respiration rates in mitochondria from rat kidney. Respiration rates in state 3, state 4 and maximal respiration rates were determined in isolated mitochondria using an oxygen electrode. Duplicate measurements were performed on each preparation and averaged. Values are means ± S.E.M. from 5 independent preparations for each group. ^∗, ∗∗^ *P* < 0.05, *P* < 0.01  compared with controls in the same condition.

The basis for this study was the observation that ageing rats exposed to maternal protein restriction *in utero *exhibit glomerular injury and loss of renal function, both of which are possibly mediated by an imbalance of reactive oxygen species and antioxidant protection [12, 13]. Mitochondria are the primary source of reactive oxygen species, so functional studies of mitochondria isolated from kidneys of control and protein-restricted offspring, were performed. As shown in Figure 1, mitochondrial respiration in kidneys from adult rats was reduced by maternal protein restriction. State 3 and state 4 respiration rates were approximately one-third lower in offspring of LP dams compared to the offspring of control fed dams (*P* < 0.01). The maximal respiratory rate for the renal mitochondria was reduced by 17% in the LP group. State 3 represents the actively respiring state, whilst state 4 describes respiration in the absence of ATP synthesis. The data are consistent with a general downregulation of mitochondrial function. Decreased mitochondrial respiration is associated with a number of disease states, including metabolic disturbance associated with obesity. Essop and colleagues reported that in hearts of young, obese, prediabetic rats, state 4 respiration was reduced, and the capacity of isolated mitochondria to recover state 3 respiration following oxygen depletion was impaired [29]. It is known that rats exposed to maternal protein restriction in fetal life develop insulin resistance and glucose intolerance with ageing [9, 28], so the current observation of impaired mitochondrial respiration is of interest in this context. It was also noted that although maximal respiratory rate declined as did state 4, the state 3 respiratory rate declined further, leaving a greater reserve capacity and a lower respiratory control ratio. This could be of importance with regard to the programmed animals' ability to cope with challenges, possibly suggesting an inappropriate adaptation in an attempt to align supply and demand. Further studies need to address the ability of LP exposed animals to cope with challenges to their regulation of bioenergetics.

**Figure 2:**
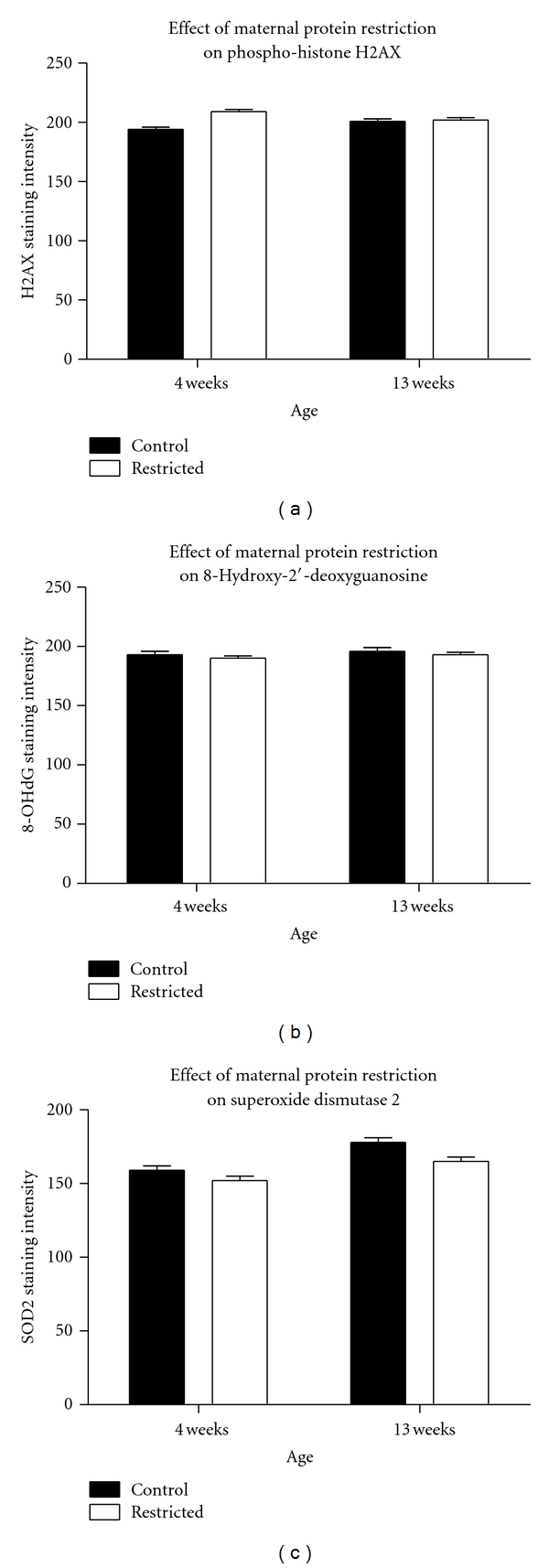
Oxidative stress in kidney tissue as measured by immunohistochemistry. Markers of oxidative stress within the mitochondria were unaffected by maternal protein restriction. Values are means ± S.E.M. *n* = 4–6.

No other studies have determined the effects of maternal nutrition upon mitochondrial respiration and its regulation by alterations in proton conductance. The reduced rate of respiration that we observed could appear to contradict the assertion that altered mitochondrial respiration results in greater release of reactive oxygen species, causing more oxidative injury to the tissues. This interpretation was further supported by the observation that maternal protein restriction did not impact upon any of the markers of oxidative stress that were measured (Figure 2). Protein expression of H2AX and SOD2 were unaffected by maternal protein restriction, and there was no evidence of increased 8-OHdG. The lack of evidence of oxidative processes may be indicative of mitochondrial reactive oxygen species production playing no role in renal injury associated with maternal protein restriction. Alternatively, it may be the case that oxidative injury occurs much later in life. Joles et al. found little evidence of oxidative damage in kidneys of rats exposed to prenatal protein restriction, aged under 18 months, although proteinuria and declining function occurred in much younger animals [12, 13]. It may be that what is being observed here is that these programmed animals successfully adapted their respiratory function to cope with their current environment, which when compared to control animals appears to be early mitochondrial dysfunction. It only develops into a potentially damaging process with ageing, when they are unable to cope with decreasing cell number. It is possible that the time points selected in this study were too early, but clearly a bioenergetics approach is of utility as it appears to reveal changes that occur prior to tissue damage and to offer some predictive power. The methods used in this study could be used to test interventions in programmed animals that have the potential to prevent or delay age-related disease. It would be highly desirable to establish noninvasive biomarkers of mitochondrial function, for example, suppressed serum coenzyme Q10 or elevated urinary concentrations of Krebs cycle intermediates, which would enable findings of such studies to more readily translate into human interventions.

**Figure 3:**
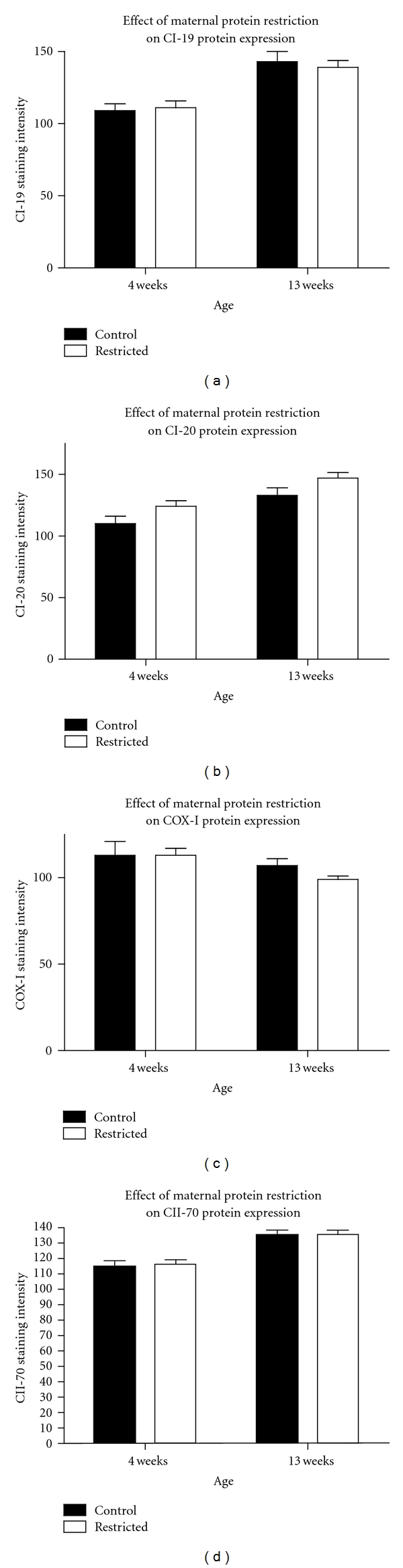
OXPHOS pathway proteins in kidney tissue as measured by immunohistochemistry. Protein expression of key subunits from complexes I and IV were unaffected by maternal protein restriction. Values are means ± S.E.M.  *n* = 4–6.

**Figure 4:**
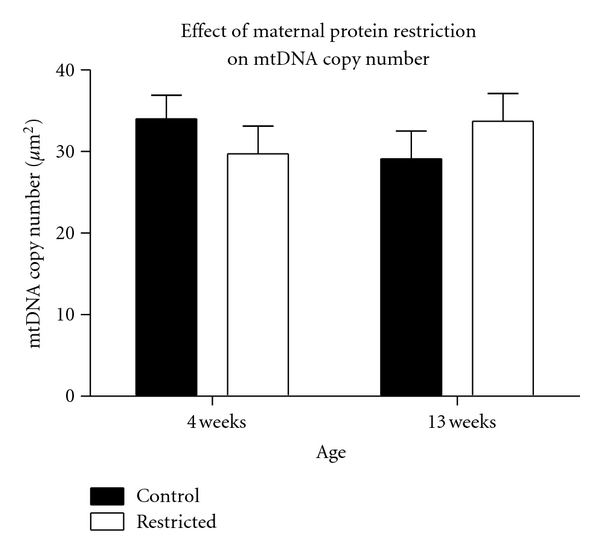
Mitochondrial DNA copy number in kidney as determined by PCR. mtDNA copy number was unaffected by maternal protein restriction. Values are means ± S.E.M. *n* = 10.

The observed reduction in mitochondrial respiration could be suggestive of deficits in expression or activity of the electron transport chain. Mitochondrial control theory proposes that the level of electron transport chain activity must fall below critical thresholds before any deficits of mitochondrial membrane potential, oxygen consumption, or ATP synthesis can be observed [30]. Lower state 3 respiration of the low-protein exposed animals could possibly be associated with lower complex IV activity. However, COX/SDH histochemistry showed no indication of complex IV deficiency in any of the kidney tissues at 4 or 13 weeks of age in offspring of either control or protein restricted dams (data not shown). Sections stained consistently for complex IV, with no blue areas which would indicate a lack of COX activity. Similarly, immunohistochemistry indicated that maternal protein restriction had no effect on the protein expression of CI-19, COX I, or CII-70 in the kidney (Figure 3). Expression of CI-20 was slightly increased by maternal protein restriction (*P* = 0.022). However, when variability of CII-70 was used to adjust the data, this increase was no longer significant (*P* = 0.097). The data, therefore, indicate no gross impact of the maternal diet upon expression of the electron transport chain, but this does not exclude the possibility that activity of complexes I-III may be impaired. Although there was some variation in renal mitochondrial DNA copy number with age, maternal protein restriction had no effect on copy number at either 4 or 13 weeks of age (Figure 4). This suggests that differences in respiration rates were unrelated to variation in mitochondrial yields from the kidneys. Taylor and colleagues reported that maternal diet impacted upon mitochondrial copy number in rat kidneys [18]. This, however, was evidence of programming related to over- rather than under-nutrition, suggesting that the mechanistic basis of such programming may vary depending upon the nature of the dietary insult. Mitochondrial copy number and the expression of the mitochondrial genes decline with ageing [31]. In keeping with the observation that oxidative injury develops with age [13], it may be hypothesized that maternal protein restriction could impact upon the ageing process, bringing about an imbalance of oxidative and antioxidant processes. It is well established that exposure to maternal protein restriction shortens lifespan and increases markers of cellular ageing [13, 15, 32].

**Figure 5:**
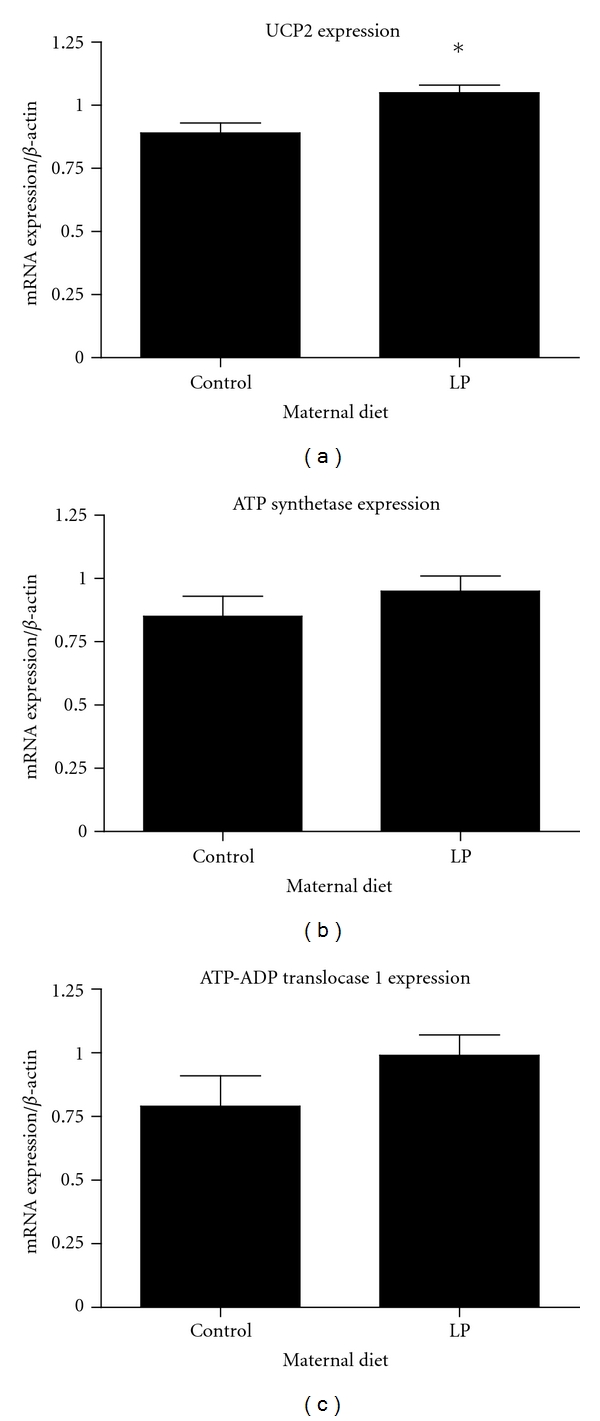
Gene expression of mitochondrial targets measured by RT-PCR. mRNA expression UCP2 was increased in offspring of protein-restricted dams. ATP Synthetase *β*-subunit and ATP-ADP translocase 1 were unaffected by maternal protein restriction. Values are means ± S.E.M. *n* = 5 animals per group.*P < 0.05 compared with control.

**Figure 6:**
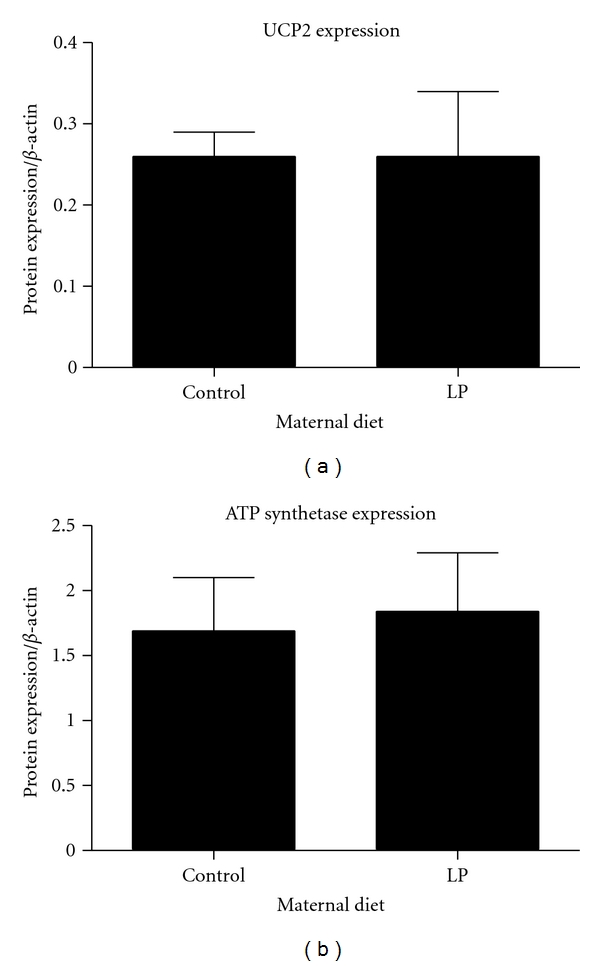
Expression of UCP2 and ATP synthetase *β*-subunit, determined by Western blot. Protein expression of UCP2 and ATP Synthetase *β*-subunit were unaltered by maternal protein restriction. Values are means ± S.E.M. *n* = 5 animals per group.

**Figure 7:**
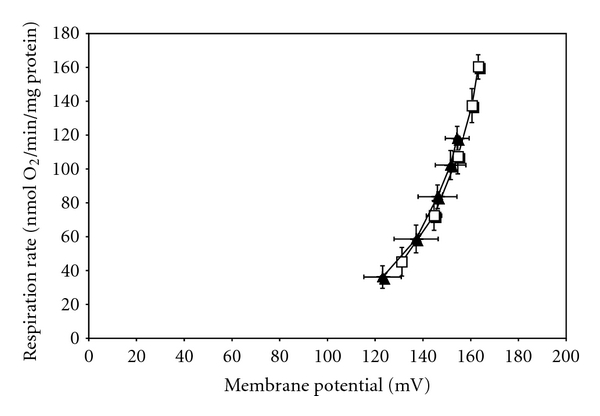
Proton leak of mitochondria from kidney showing controls and low protein fed rats. Empty box □: control; filled triangle ▲: low-protein. The highest common potential was 154 mV. Duplicate measurements were performed on each preparation and averaged. Values are means ± S.E.M. from 5 independent preparations for each group.

Lower state 3 respiration may also be associated with impaired ATP synthesis. To assess this as a potential target for nutritional programming, we determined the mRNA expression of ATP synthetase and ATP-ADP translocase 1, which is one of the proteins responsible for movement of ATP and ADP across the innermitochondrial membrane. ATP synthetase *β*-subunit and ATP-ADP translocase 1 mRNA expression were unaffected by maternal protein restriction (Figure 5). Uncoupling protein 2 allows protons to cross the inner mitochondrial membrane without ATP synthesis and reduces the mitochondrial membrane potential. In some tissues, UCP2 reduces oxidative stress [33], which corresponds to the theory that mild uncoupling can protect against ROS production becoming excessive at high membrane potentials [34]. Reduction of mitochondrial respiration may be associated with inhibition of ATP synthesis. The lower state 4 respiration of the offspring of protein restricted dams is, however, suggestive of reduced uncoupling, particularly as there was no evidence of altered expression of ATP synthetase. We found that expression of UCP2 mRNA was significantly increased in offspring of protein restricted dams (Figure 5). The difference in the expression of UCP2 mRNA was not, however, reflected at the protein level (Figure 6). In the absence of clear and consistent evidence of UCP2 overexpression, further experiments are required to assess the possibility that maternal diet impacts upon mitochondrial respiration through this mechanism. Gnanalingham and colleagues have reported that, in sheep, maternal undernutrition increases the fetal expression of UCP2 [35]. This is consistent with the notion that the programming effect is to tighten coupling to improve efficiency and what is seen when looking at the mitochondria after living on a normal diet is the compensated state achieved by increased uncoupling. When we measured membrane potential and proton leak (Figure 7), it was evident that the LP animals were operating at a lower membrane potential (state 4) but consuming the same amount of oxygen at all common membrane potentials observed. This seems consistent with what would be expected if they had been programmed to have a tightened coupling efficiency (higher membrane potential with lower oxygen consumption) but then uncoupled in response to the plentiful food supply. This would presumably be at the cost of being able to further uncouple in challenging situations where the control animals would still be able to adapt. Further research needs to be done to explore how the programmed animals differ from control animals in response to specific challenges at this level of regulation.

The present study has identified a programmed deficit of mitochondrial function in the kidney following maternal undernutrition. Whilst the evidence indicates a potential role for UCP2 in mediating this effect, the work did not have the scope to examine the mechanistic basis of the programming effect. It is widely believed that nutritional programming during early life is driven by changes to the epigenome [36]. There are a growing number of reports that maternal protein restriction can impact upon DNA methylation and histone modifications [37–39]. Although it was originally thought that the mitochondrial genome was not subjected to this level of control, it has recently been demonstrated that there is a mitochondrial DNA methyltransferase that can methylate cytosine residues [40]. Mitochondrial metabolism also determines the wider availability of S-adenosyl methionine for DNA methylation [41]. An alternative to epigenetic determination of maternally programmed effects involves overexposure of fetal tissues to glucocorticoids [11]. Undernutrition is known to suppress expression of placental 11*β*-hydroxysteroid dehydrogenase, allowing greater placental transfer of glucocorticoids from mother to fetus [42]. In sheep, early exposure to glucocorticoids upregulates expression of UCP2 [43]. Glucocorticoids may also impact upon DNA methylation [44].

## 4. Conclusion

This study has provided evidence that exposure to maternal undernutrition is associated with a decrease in mitochondrial respiration in kidneys of adult rats. Although the finding of increased expression of UCP2 mRNA could not be duplicated at the protein level, possibly reflecting changes in translation or turnover,results from these experiments suggest that early undernutrition may increase mitochondrial uncoupling under normal conditions, resulting in limited ability to effectively adjust respiratory function as cell mass declines with aging. The findings of the study are consistent with earlier reports that mitochondria may play a role in the early-life programming of adult disease. Further studies are required to assess the relative contribution of uncoupling to programming of physiological function in a broad range of tissues.
